# Insertion of Intrauterine Devices (IUDs) in Sanctuary Chimpanzees (*Pan troglodytes*) Using Ultrasonography and Radiology to Confirm Placement

**DOI:** 10.1111/jmp.70036

**Published:** 2025-10-08

**Authors:** Jenny E. Jaffe, Nancy Sokkary, Jill Nielsen, James R. McClearen

**Affiliations:** ^1^ Project Chimps Morganton Georgia USA; ^2^ Department of Veterinary Medicine Freie Universität Berlin Germany; ^3^ Department of Obstetrics and Gynecology, Children's Healthcare of Atlanta/Emory School of Medicine Atlanta Georgia USA

**Keywords:** breeding, contraception, great apes, non‐human primates, speculum

## Abstract

**Background:**

Reproduction in captive chimpanzees ( *Pan troglodytes* ) can be controlled by the insertion of intrauterine devices (IUDs) for females who do not reliably take oral contraceptives. Previous literature describes the use of improvised devices made from syringe cases as a speculum to accommodate the depth of the vaginal vault.

**Methods:**

Copper IUDs (model T380A) were inserted in two sanctuary‐housed chimpanzees. A disposable human small‐sized vaginal speculum (Welch Allyn KleenSpec) with an illumination system provided a good view of the cervix. A 3/4 mm dilator and a uterine sound aided insertion.

**Results:**

After multiple rounds of dilation, the uterine sound was still necessary to manipulate the IUD to the fundus of the uterus. Correct placement was confirmed by ultrasonography and radiology.

**Conclusion:**

Inserting IUDs in chimpanzees can prove challenging. Having appropriate equipment available, such as varied sizes of speculums and dilators, is crucial. Gynecologists and ultrasonographers with experience inserting IUDs in humans can help ensure correct placement.

## 1 Introduction

Intrauterine devices (IUDs) may be chosen as the best contraceptive option for captive female chimpanzees ( *Pan troglodytes* ) who do not take oral contraceptives dependably, as other methods all have drawbacks relating mainly to availability, effectiveness, social consequences, practicality, side effects, reversibility, and level of invasiveness [1]. The cost of acquiring an IUD commercially can vary significantly by location; for the copper 380A model, approximately $1200 USD in the US, $100 USD in Europe/Australia, and much less in Asia and Latin America [2]. This model should be effective for 10–12 years [3], or even up to 15–20 years [4], meaning it can be more economical than oral contraceptives over time. Studies report [5, 6] non‐significant levels of expulsion with older models, especially in younger, nulliparous chimpanzees, but this has not been the case when using the Paragard IUD (model T380A) in fully adult laboratory chimpanzees in recent decades (Dana Hasselschwert, personal communication, November 18, 2024). In humans, some studies report decreased hemoglobin levels for users of copper IUDs [7, 8], though this has not been observed in chimpanzees (Dana Hasselschwert, personal communication, September 9, 2025). A rare possible complication is migration of the IUD from the uterus, for example, to the bladder [9]. Previous reports describing the use of IUDs in captive chimpanzees describe the use of improvised devices made from syringe cases as a speculum to accommodate the depth of the vaginal vault, which can be up to twice as long as in humans [6, 9, 10]. This report aims to provide a brief update to the available literature, showing how ultrasonography and radiology help confirm placement, and describing the use of a disposable human speculum (which allows for the placement of a cordless illumination device) and troubleshooting a small cervical os.

## 2 Materials and Methods

A 20‐year‐old (AF1) and a 14‐year‐old (AF2) female chimpanzee took oral contraceptives sporadically since arriving at Project Chimps, a chimpanzee sanctuary for former laboratory chimpanzees. Compared to the other chimpanzees, they were suspicious whenever oral medications were added to their normal treats (juice, peanut butter, or protein shakes). Neither chimpanzee had any known health concerns. They tested negative for pregnancy by human hCG tests (First Sign, Hemosure) on urine collected after witnessing urination, followed by pipetting the urine from the floor of the enclosure.

Both chimpanzees were anesthetized for routine physical examinations using a combination of tiletamine‐zolazepam, ketamine (both intramuscular), propofol (intravenous), and isoflurane (by inhalation), AF1 on January 31, 2025, and AF2 on May 16, 2025. After the routine physical examination, the IUDs were inserted.

The chimpanzees were placed in dorsal recumbency, with a foam wedge under the pelvis and the legs retracted cranially to allow optimal access to the vagina. A clear view of the cervix was obtained using a clear acrylic KleenSpec disposable speculum, size small (Welch Allyn). Sizes available are (length × width in cm): extra small (15.34 × 1.63), small (17.63 × 2.24), medium (17.63 × 2.94), large (19.43 × 3.56), with more detailed measurements available online (Welch Allyn). All sizes of this model allow for the insertion of a cordless illumination device in the handle of the speculum, though in AF1 a headlamp was used as an alternative, as there was a malfunction of the illumination device's battery on the day of the procedure. The medium‐ and large‐sized speculum proved too wide for both chimpanzees. The same was true for the improvised speculum made with a 60 mL syringe case (Monoject, Covidien) as described in Hasselschwert & Fontenot [10].

The cervix and vagina were prepped with povidone‐iodine. A ring forceps was used to grasp a povidone‐iodine swabstick (McKesson) to accommodate the length and circumference of the vagina. An Allis clamp (Sklar Allis Tissue Forceps 9–1/2 in. straight 5 × 6 teeth stainless steel) was then used to grasp the anterior lip of the cervix. Subsequently, a uterine sound (Medline‐Uterine Sounds Sims‐Mall 12–1/2 in. length, curved rigid tip) was used under transabdominal ultrasound guidance (Mindray M7 portable ultrasound machine) to dilate the cervix and in AF2 to measure the length of the uterine cavity.

For AF1, an initial attempt was made to insert the copper Paragard IUD (model T380A), but the circumference of the insertion device was noted to be too large for the cervical os. Instead, the IUD was loaded into the insertion device in the typical manner, tucking the two arms into the insertion device. The insertion device was advanced to be flush with the external os, such that the tip of the IUD was within the cervical canal. The plunger was engaged to advance the IUD into the lower uterine segment. Under ultrasound guidance, the uterine sound was then used to manipulate the IUD to the fundus of the uterus (verified on ultrasound).

For AF2, the equivalent model T‐SAFE CU 380A QL was used. Here the circumference of the insertion device initially seemed to be too large as well, but after multiple rounds of dilation with a 3/4 mm double‐ended dilator and the uterine sound, the insertion device could be introduced into the os. After removal of the plunger, on ultrasound the IUD did not appear to have been fully advanced, so again, the uterine sound was used to push the IUD towards the fundus of the uterus until the correct position was confirmed under ultrasound guidance.

The strings were cut to a length 2 cm distal to the cervix and the Allis clamp was removed. As the final step of the procedure, the cervix and vagina were evaluated, and only very minimal bleeding was noted.

## 3 Results

**FIGURE 1:**
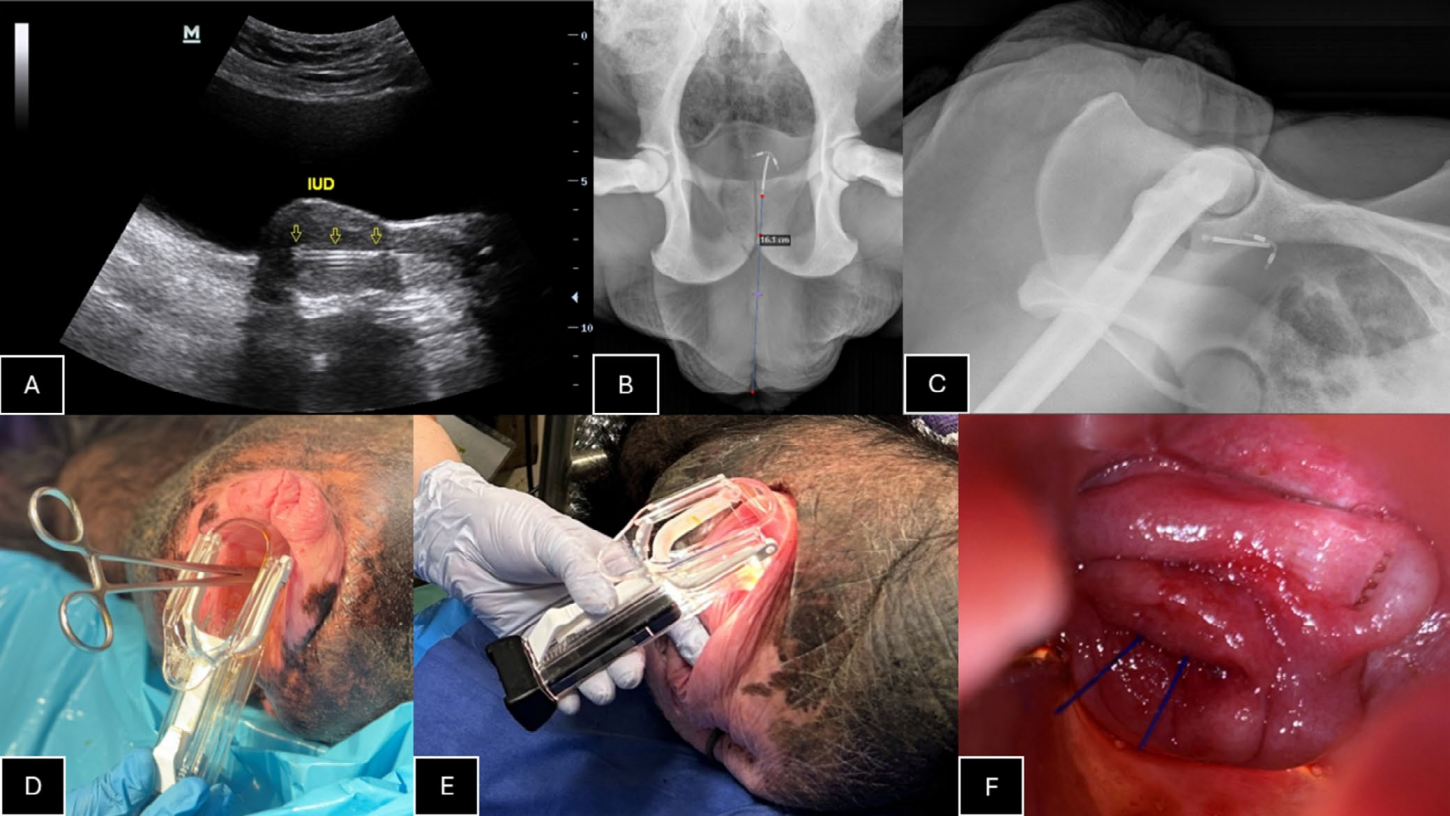
Ultrasonography showing IUD in uterus in AF1 (A), ventrodorsal and lateral pelvic radiograph in AF1 (B, C), disposable speculum in use without illumination in AF1 and with illumination in AF2 (D, E), cervical os with shortened strings of IUD visible in AF2 (F).

Successful placement was confirmed during the insertion by ultrasound imaging (see Figure 1A) and after insertion by ventrodorsal and lateral pelvic radiographs (see Figure 1B,C). The distance from the internal cervical os to the vaginal opening on the ventrodorsal pelvic radiograph was approximately 16 cm for both females (see measurement for AF1 in Figure 1A); for AF2, uterine depth was 8 cm, as measured by the uterine sound. The chimpanzees recovered from anesthesia uneventfully and with no obvious discomfort or bleeding in the days after the procedure.

## 4 Discussion

There is a known variability in vaginal depth, between 11 and 22 cm [9]. In both chimpanzees, the depth was roughly average at 16 cm; however, these measurements are approximations due to being taken from radiographs. Using a human speculum size small provided a better view of the cervix than the size medium or large or the improvised speculum due to the relatively narrow vaginal canal. In our experience (unpublished data from cervical exams in anesthetized chimpanzees), the Welch Allyn KleenSpec speculum in size medium or large is normally adequate for most adult chimpanzees, with the added benefit of the illumination device, which provides better lighting than the current alternative, the headlamp. We would recommend having three sizes (small, medium and large) of the disposable human speculum on hand with the improvised syringe casing speculum in several sizes (60, 35 and 20 mL) available as a backup.

The cervix, in both cases, was noted to be small and narrow. As a result, the insertion device of the copper IUD (model 380A) could not be introduced into the uterine cavity in AF1. It was possible to advance the IUD to the lower uterine segment/cervix and then use a sound to advance it to the correct fundal location (see images). This could not be done blindly, and ultrasound guidance was imperative. For AF2, repeated gentle dilation of the cervix eventually allowed the insertion device to be introduced, but the sound was still necessary to ensure correct placement under ultrasound guidance. IUD insertion devices range in diameter from 3.8 to 4.8 mm [11]. However, given that the copper IUD is the only option that is hormone‐free with an efficacy of 10–12 years [3], it is likely the best option despite the slightly larger inserter. Having larger, graduated sizes of dilators available (5/6 mm and up) would likely aid insertion.

The ultrasonography stills and radiology images included may help those inserting IUDs in chimpanzees for the first time, or those checking chimpanzees with existing IUDs for the current position of the device. In some chimpanzees that are trained to allow conscious abdominal ultrasonography, or conscious radiography [12], this could provide an excellent opportunity for verification of the IUD placement without additional anesthesia.

## 5 Conclusion

Inserting IUDs in chimpanzees can prove challenging [6, 9, 10]. Having the appropriate equipment available, such as varied sizes of speculums and dilators, is crucial. Gynecologists and ultrasonographers with experience inserting IUDs in humans can be helpful. If the veterinarian has never performed the procedure before, reviewing the available literature on IUD insertion in chimpanzees is critical.

## Ethics Statement

The authors confirm that the ethical policies of the journal, as noted on the journal's author guidelines page, have been adhered to. Ethical approval was not required because no animals were used for research in this study; rather, this report covers using standard of care contraceptive treatment.

## Conflicts of Interest

The authors declare no conflicts of interest.

## Data Availability

The data that support the findings of this study are available from the corresponding author upon reasonable request.
